# Lack of Functional Trehalase Activity in *Candida parapsilosis* Increases Susceptibility to Itraconazole

**DOI:** 10.3390/jof8040371

**Published:** 2022-04-05

**Authors:** Ruth Sánchez-Fresneda, María Luz Muñoz-Megías, Genoveva Yagüe, Francisco Solano, Sergi Maicas, Juan Carlos Argüelles

**Affiliations:** 1 Área de Microbiología, Facultad de Biología, Universidad de Murcia, 30100 Murcia, Spain; ruth.sanchez1@um.es (R.S.-F.); luzmegias19@yahoo.es (M.L.M.-M.); 2 Servicio de Microbiología Clínica, Hospital Universitario Virgen de la Arrixaca, IMIB, 30331 Murcia, Spain; gyague@um.es; 3 Departamento de Bioquímica y Biología Molecular B e Inmunología, Facultad de Medicina, Universidad de Murcia, 30100 Murcia, Spain; psolano@um.es; 4 Departament de Microbiologia i Ecologia, Facultat de Biologia, Universitat de València, Burjassot, 46100 València, Spain

**Keywords:** fluconazole, itraconazole, ROS, mitochondrial activity, trehalase, trehalose, *Candida parapsilosis*

## Abstract

Central metabolic pathways may play a major role in the virulence of pathogenic fungi. Here, we have investigated the susceptibility of a *Candida parapsilosis* mutant deficient in trehalase activity (*atc1*Δ/*ntc1*Δ strain) to the azolic compounds fluconazole and itraconazole. A time-course exposure to itraconazole but not fluconazole induced a significant degree of cell killing in mutant cells compared to the parental strain. Flow cytometry determinations indicated that itraconazole was able to induce a marked production of endogenous ROS together with a simultaneous increase in membrane potential, these effects being irrelevant after fluconazole addition. Furthermore, only itraconazole induced a significant synthesis of endogenous trehalose. The recorded impaired capacity of mutant cells to produce structured biofilms was further increased in the presence of both azoles, with itraconazole being more effective than fluconazole. Our results in the opportunistic pathogen yeast *C. parapsilosis* reinforce the study of trehalose metabolism as an attractive therapeutic target and allow extending the hypothesis that the generation of internal oxidative stress may be a component of the antifungal action exerted by the compounds currently available in medical practice.

## 1. Introduction

Although *Candida albicans* remains the most prevalent species of *Candida* responsible for both superficial and invasive candidiasis, which mainly affects the immunodebilitated population [1,2,3], another group of opportunistic yeasts belonging to the genus *Candida* and referred to as “non-albicans” has emerged in recent years as responsible for numerous nosocomial outbreaks [3,4]. The taxonomical complex *C. parapsilosis* has an increasing clinical incidence, being the second or third most frequently isolated, depending on the geographic region studied [5,6,7]. As a matter of fact, although infections caused by *C. parapsilosis* generally have lower morbidity and mortality rates than *C. albicans*, they pose a serious threat in patients undergoing intensive surgery and in those harboring catheters and other implants due to the formation of tenacious biofilms [5,6,7,8].

A number of important pathobiological characteristics are markedly different between *C. albicans* and *C. parapsilosis*. They encompass virulence capacity, the susceptible patient groups that develop septicemic infections or mechanisms of antifungal sensitivity, and drug resistance [5,6,8,9,10]. Thus, *C. parapsilosis* is unable to form true hypha and lacks any distinctive sign of a sexual cycle [5,6,8]. Regarding antifungal treatments, there are also considerable differences depending on the outbreaks and the countries analyzed. Generally, echinocandins appear to be less effective on certain clinical isolates, while other variants are resistant to azoles [5,6,7,8,9]. This scenario constitutes a major therapeutic drawback due to the limited arsenal of antifungals currently available [11].

In the search for new antifungals, central nutritional pathways, in particular those involved in glucose metabolism, have emerged as interesting targets [12,13]. In fact, the non-reducing disaccharide trehalose has been proposed as a preferred candidate for the design of alternative antifungal therapies, considering the wide evidence collated from the highly prevalent pathogenic fungi *C. albicans*, *Cryptococcus neoformans*, and *Aspergillus* spp., among others [14,15,16]. Regarding the genus *Candida*, the trehalose biosynthetic pathway (*TPS1* and *TPS2* genes) has received preferential attention, as it is a main virulence factor involved in normal glucose growth, tissue adhesion, resistance to oxidative stress, and macrophage death or hypha formation in tps1-null mutants [17,18,19].

Trehalase activity, which is the hydrolase responsible for trehalose cleave-off, is also a component contributing to pathogenicity in *C. albicans* and *C. glabrata* [20,21,22]. In the case of *C. parapsilosis*, we have previously disrupted the two genes (*ATC1*/*NTC1*) encoding trehalase activity in this yeast [10,23]. The resulting homozygous mutant showed a severe deficiency in cell growth as well as an impaired ability to infect cells and to form consistent biofilms [23]. We have examined here the fungicidal sensitivity of this null mutant to the azoles FLC and ITC compared to its isogenic parental strain. Our results support the use of the *ATC1* and *NTC1* genes as interesting targets for the design of new antifungals.

## 2. Materials and Methods

### 2.1. Strains and Growth Conditions

The *C. parapsilosis* strains used in this study have been reported elsewhere [10,23]. Liquid cultures were grown at 37 °C by shaking in YPD medium consisting of 2% peptone, 1% yeast extract, and 2% glucose. Solid media contained 2% agar. Time-course growth in liquid medium was measured by monitoring cell density at OD_600_ or by direct cell counting in a TC-20 cell counter (BioRad, Hercules, CA, USA). Cell viability was determined in samples diluted appropriately with sterile water by plating in triplicate on solid YPD after incubation for 1–2 days at 37 °C. Between 30 and 300 colonies were counted per plate. Survival rates were normalized to control samples (100% viability). Colony growth in solid medium was tested by spotting 5 μL from the respective ten-fold dilutions onto YPD agar. Then, the plates were incubated at 30 °C and scored after 24 or 48 h.

### 2.2. Preparation of Cell-Free Extracts

After exposure to different stresses, samples from the cultures were harvested and resuspended at known densities (10–15 mg/mL, wet weight) in the extraction buffer, 100 mM 4-morpholine-ethanesulfonic acid (MES) pH 6.0, containing 5 mM cysteine and 0.1 mM phenyl methyl sulphonyl fluoride (PMSF). Cellular suspensions were transferred into small pre-cooled tubes (1.0 cm diameter) with 1.5 g Ballotini glass beads (0.45 mm diameter). The cells were broken by vigorously vibrating the tubes in a vortex mixer. The tubes were rapidly cooled in ice. Then, the crude extract was centrifuged at 10,000× *g* for 5 min, and the pellet was resuspended in the same buffer at the initial density. For antioxidant assays, the supernatant fraction obtained was filtered through Sephadex G-25 NAP columns (Amersham Pharmacia Biotech AB, Staffanstorp, Sweden) previously equilibrated with 50 mM K-phosphate buffer, pH 7.8, to remove low-molecular-weight compounds.

### 2.3. Enzymatic Assays

Acid trehalase was measured by incubating 50 μL of cell-wall pellet with 200 μL of 200 mM trehalose prepared in 200 mM sodium citrate pH 4.5 containing 2 mM EDTA. The reaction for neutral trehalase activity contained 50 μL of cell-free extract (25–100 μg of protein) and 200 μL of 200 mM trehalose prepared in 25 mM MES pH 7.1, 125 μM CaCl_2_. The assay mixtures were incubated at 30 °C for 30 min and stopped by heating in a water bath at 100 °C for 5 min. The glucose released was determined by using the glucose oxidase–peroxidase method. The specific activity is expressed as nmol of glucose released min^−1^ mg of protein^−1^.

### 2.4. ROS and Membrane Potential Determination by Flow Cytometry

Intracellular ROS formation by flow cytometry with dihydrofluorescein diacetate (DHF) was measured following the procedure described in [24] with the additional modifications indicated elsewhere [23]. Mitochondrial membrane potential was also determined by flow cytometry using Rhodamine 123 as fluorochrome. DHF and Rhodamine 123 were added to the samples at a final concentration of 40 μM and 20 μM, respectively, and incubated at 37 °C for 30 min. After treatment with Rhodamine 123, the cells were washed twice with PBS to remove excess of fluorochrome. Fluorescence intensity was determined using the EPICS XLMCL4 cytometer (Beckman Coulter, Nyon, Switzerland) equipped with an argon ion laser with an excitation power of 15 mW at 488 nm. Forward scatter (FSC) and side scatter (SSC) were analyzed on linear scales, while the analyses of green (FL1) fluorescence intensity were made on a logarithmic scale. Analysis gates were set around debris and intact cells on an FSC vs. SSC dot plot. Fluorescence histograms corresponding to 5000 cells were generated using the gated data. Data acquisition and analysis were performed using WINMDI software (available from http://facs.scripps.edu, accessed on 13 January 2022).

### 2.5. Biofilm Formation

*C. parapsilosis* biofims were obtained in vitro on the surface of 96-well polystyrene microtiter plates as described previously [25]. Briefly, 100 μL of the standardized mutants of *C. parapsilosis* suspension (1 × 10^6^ blastoconidia/mL) in RPMI 1640 was allowed to adhere and form biofilms at 37 °C for 24 h. After biofilm formation, the medium was aspirated, and non-adherent cells were removed by washing three times with sterile PBS. Quantification of biofilms was performed by (3-(4,5-dimethyl-2-thiazolyl)-2,5-diphenyl-2H-tetrazolium bromide) (MTT, Sigma Chemicals, Saint Louis, MO, USA) reduction assay. MTT was prepared as a saturated solution at 0.5 g L ^−1^ in PBS, filter-sterilized though 0.22 μm pore-size filters, aliquoted, and stored at −70 °C. An aliquot of the MTT stock solution was thawed prior to each assay, and 10 mM menadione (Sigma Chemicals, Saint Louis, MO, USA) in acetone was added to give a final concentration of 25 μM. An aliquot of 100 μL of the MTT–menadione solution was added to each well, and the plates were incubated for 2 h at 37 °C. The metabolic activity of sessile *C. parapsilosis* cells was assessed quantitatively by measuring absorbance in a microtiter plate reader (Asys Jupiter) at 540 nm. The tetrazolium salt that accumulated after MTT reduction by cellular dehydrogenases was proportional to the number of viable cells present in the biofilm.

## 3. Results

### 3.1. Cell Survival Rate of *C. parapsilosis* after Treatment with Azoles

In *C. parapsilosis*, a single gene encodes both enzymes called neutral trehalase (*CpNTC1*) and acid trehalase (*CpATC1*) [10,23]. Enzymatic assays revealed the lack of any detectable trehalase activity (lower than 0.3 units/mg protein) during the growth cycle on a glucose-containing medium (YPD) of a homozygous *C. parapsilosis* null mutant *atc1*Δ/*ntc1*Δ (Table 1), obtained by the simultaneous disruption of the two individual genes [23].

These results provide further support on the inability of these mutant cells to mobilize endogenous trehalose or to hydrolyze exogenous disaccharide during their growth cycle [23]. Then, the corresponding MICs values for both the parental and *atc1*Δ/*ntc1*Δ strains were calculated as 0.5 μg/mL for fluconazole (FLC) and 0.15 μg/mL for itraconazole (ITC), following the EUCAST protocol. The susceptibility of *C. parapsilosis* to these two azolic compounds has been chosen as the main objective of this study.

**Figure 1:**
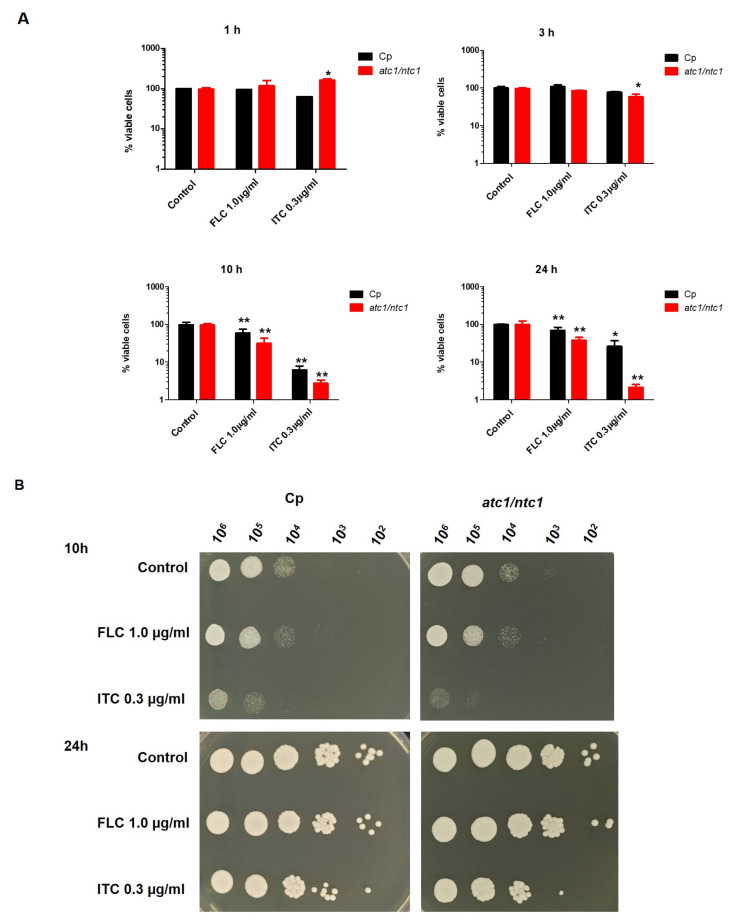
Time-course effect of FLC and ITC on cell survival recorded in the parental strain of *C. parapsilosis* (*Cp*) and the trehalase-deficient null mutant *atc1*Δ/*ntc1*Δ. YPD-grown cultures of the two strains were exposed to the indicated doses of antifungals and incubated at 37 °C. Identical samples (10^7^ cells/mL) were harvested at the indicated times. (**A**) Viability in liquid medium was determined after appropriate dilution with sterile water by plating in triplicate on solid YPD and incubating for 2–3 days at 37 °C. A control sample was left without treatment (100% viabilily). The experiment was repeated twice with similar results, and the values shown are the mean ± standard deviation of three independent determinations. The difference between the mean values obtained was statistically significant at *p* < (0.05) (*), *p* < (0.01) (**) according to the Mann–Whitney range test. (**B**) Ten-fold cell suspensions containing the specific compound were spotted (5 μL) on YPD plates, which were scored after 48 h.

The potential toxic effect triggered by the addition of ITC and FLC has been determined in YPD-grown active cells of the *C. parapsilosis* parental strain and the isogenic trehalase-deficient null mutant. Considering that azoles are mainly fungistatic compounds, a time-length quantification of viable cells was monitored upon the application of 1.0 μg/mL FLC (2 × MIC) and 0.3 μg/mL ITC (2 × MIC). Lower doses did not cause a significant decrease in cell viability. As shown in Figure 1A, exposure to these antifungal concentrations had virtually no effect after 1 h, and only a gradual dose-dependent reduction in the level of viable cells could be recorded (Figure 1A). This loss was only evident after 10 h of incubation in the two cell types, being more pronounced in *atc1*Δ/*ntc1*Δ cells and maintained until 24 h, whereas at this time, the viability of parental cells had largely been recovered (Figure 1A). A similar profile of azole susceptibility was confirmed by spotting 10-fold cell suspensions containing the antifungal on solid YPD plates (Figure 1B). For the sake of clarity, only aliquots taken at 10 and 24 h are shown. As can be seen, an inhibitory effect of ITC on colonial growth was evident after 10 h of treatment, whereas at 24 h, a remarkable recovery was evident (Figure 1B).

### 3.2. Level of ROS Formation and Mitochondrial Activity after Addition of Fluconazole and Itraconazole

Induction of internal oxidative stress acts as an additional component of the fungicidal effect triggered by some antifungals, i.e., Amphotericin B, against highly prevalent pathogenic yeasts such as *Candida* species, although this mechanism does not appear to be operative with other agents examined, such as Micafungin [26,27]. However, the universal validity of this factor has yet to be extended to other members of the antibiotic and drugs families endowed with antifungal activity, as well as against other important infectious fungi, such as *Aspergillus* or *Cryptococcus* [24,28,29].

**Figure 2:**
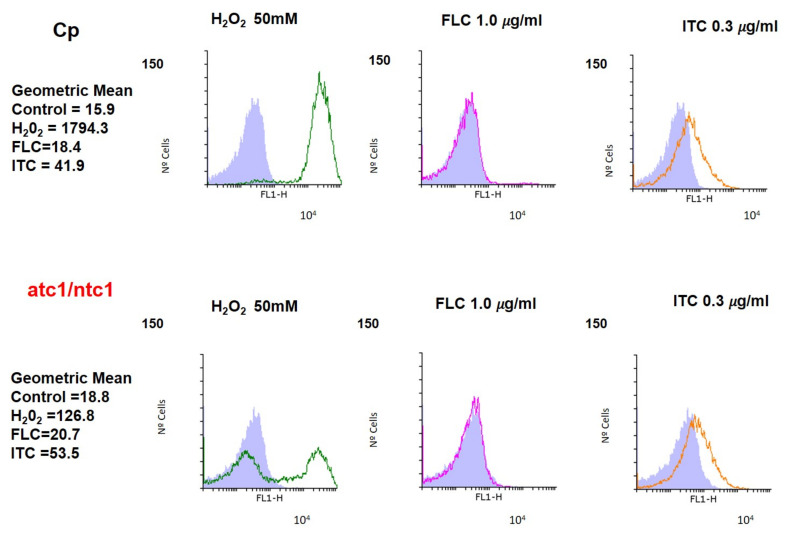
Endogenous ROS formation after the addition of FLC (red histograms) and ITC (orange histograms). Exponential yeast-like cells of the two *C. parapsilosis* strains were grown in YPD medium overnight, resuspended in PBS buffer, and then treated with the two antifungals for 1 h at 37 °C. H_2_O_2_ 50 mM (green histograms) was introduced as a positive marker for oxidative stress. The samples were analyzed with dihydrofluorescein (DHF) by flow cytometry as described in Methods. Control assays correspond to the gray area, and identical aliquots were treated with 1.0 μg/mL FLC and 0.3 μg/mL ITC.

**Figure 3:**
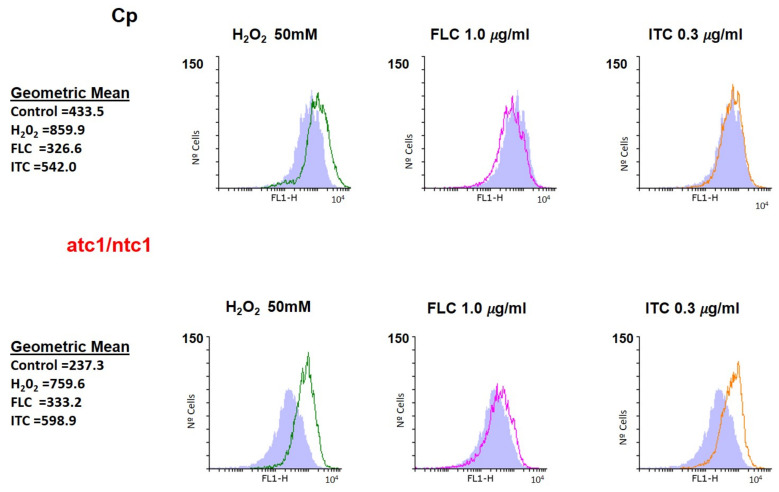
Determination of mitochondrial membrane potential after treatment with FLC and ITC. Equivalent cell samples harvested from actively growing cells were treated with the azoles for 1 h at 37 °C and immediately analyzed by flow cytometry using Rhodamine 123 (20 μM) as described in Methods. For other details, see Figure 2.

Since the information on this topic in the case of azoles is inconclusive, we monitored both the endogenous production of ROS by staining with DHF and the mitochondrial membrane potential with rhodamine (see Methods) in YPD-exponential cultures of the two *C. parapsilosis* strains, which were treated for 1 h with ITC and FLC. A positive oxidative stress control (H_2_O_2_ 50 mM) was included in the assays (Figure 2 and Figure 3). As shown in Figure 2, upon the addition of H_2_O_2_, the endogenous levels of ROS increased significantly in the parental and *atc1Δ/ntc1ΔC. parapsilosis* strains, although the fraction of cells able to produce ROS was clearly lower in the mutant. However, exposure to FLC did not induce any relevant change in the basal level of ROS compared to the control sample (gray area) in the two strains analyzed (Figure 2). In contrast, the addition of 0.3 μg/mL ITC caused a clear rise in the intracellular content of ROS, with no appreciable differences between parental and mutant cells (Figure 2).

The data obtained from the simultaneous determination of mitochondrial activity are presented in Figure 3. The positive control (H_2_O_2_) showed a weak increase in membrane potential, which was slightly higher in the *atc1*Δ/*ntc1*Δ mutant. Remarkably, the alterations caused in the mitochondrial activity of parental cells by the presence of ITC were practically negligible, whereas in the *atc1*Δ/*ntc1*Δ cells, a marked increase could be recorded (orange area) (Figure 3). As occurred with ROS, the addition of FLC had an undetectable effect on the mitochondrial membrane potential (Figure 3). Therefore, the toxic effect triggered by ITC on *C. parapsilosis* cells deficient in trehalase activity (Figure 1) could be explained, at least in part, by the generation of endogenous oxidative stress, which was manifested by both an increased production of ROS and mitochondrial activity (Figure 2 and Figure 3).

### 3.3. Effect of Antifungal Treatment on Trehalose Synthesis

In relevant pathogenic fungi, the endogenous accumulation of trehalose is a contributory factor of virulence [14,15,16,30] and plays a main protective role of cellular integrity against oxidative stress as well as coping with treatment with various antifungals, particularly AmB [31]. Therefore, the content of this non-reducing disaccharide has also been quantified in exponential cultures of both strains in response to specific exposure to FLC and ITC. As shown in Table 2, the basal level of trehalose was higher in the null mutant than in the parental cells, reflecting the lack of a functional trehalase (Table 1). However, only the specific addition of ITC was able to induce some increase in intracellular trehalose content, while the effect of FLC addition was weaker (Table 2).

### 3.4. Level of Biofilm Formation in Trehalase-Deficient Mutant of *C. parapsilosis*

**Figure 4:**
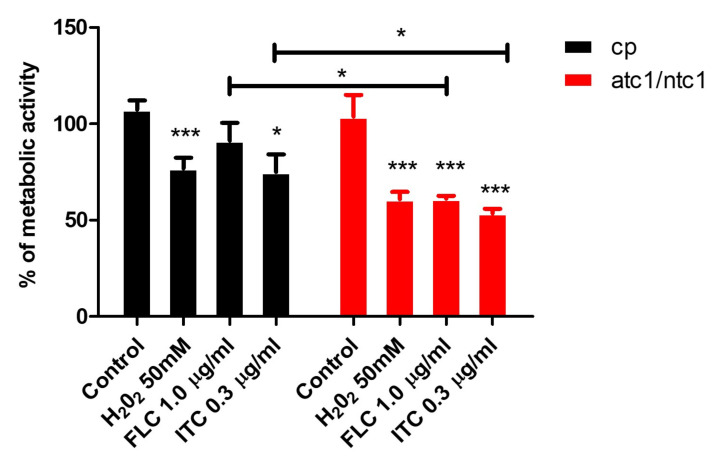
Biofilm formation by the parental strain of *C. parapsilosis* and the trehalase-deficient null mutant *atc1*Δ/*ntc1*Δ. Biofilms were preformed for 24 h in 96-well plates. At this moment, FLC, ITC, and H_2_O_2_ were added at the indicated concentrations. Metabolic activity was quantified after 24 h by XTT reduction assay. Results are expressed as mean ± standard deviation of two experiments with five replicates for each group. Statistically significant differences (* = *p* < 0.05; *** = *p* < 0.001) were recorded with respect to an untreated control according to the Mann–Whitney U test.

Infections that give rise to the formation of highly-structured stable biofilms are frequently associated to high rates of morbidity and mortality in hospitalized patients [32]. Drug-resistant biofilms have become a worrying sanitary concern, since therapeutic treatment is problematic [32,33,34]. In *C. parapsilosis*, the disruption of genes encoding trehalase activity results in a clear reduction in the ability to form biofilms [23]. According to Figure 4, when prefixed sessile cells of the two strains were incubated in the presence of FLC or ITC, they underwent a significant decrease of the biofilm-dependent metabolic activity, which was more profound in *atc1*Δ/*ntc1*Δ cells compared to the parental strain (Figure 4). Again, ITC was more effective than FLC regarding loss of biofilm formation; this difference was greater in *C. parapsilosis* cells than in mutant cells (Figure 4). An exposure to an oxidant (H_2_O_2_) was also able to induce a reduction of preformed biofilms (Figure 4). These preliminary data support the introduction of antibiotic lock therapy to eradicate active fungal biofilms that adhere on indwelling medical devices [27,32,33,34,35].

## 4. Discussion

The arsenal of currently available antifungal compounds is insufficient to deal with the dramatic increase in invasive fungal infections recorded in recent decades, which is mainly associated to the expanding immunocompromised population [1,2,3]. Furthermore, the growing isolation of strains resistant to conventional antibiotics as well as the frequent identification of nosocomial outbreaks of fungal species traditionally classified as innocuous largely complicates this scenario. In fact, the mortality rates caused by *C. albicans* in bloodstream and hospital-acquired infections can reach up to 40–50% [1,36]. Therefore, the development of novel, more potent, and safer antifungal compounds is an urgent clinical need [11].

Among other therapeutic strategies, several groups have explored the introduction of biochemical and genetic alterations in several key nutritional pathways as potentially interesting antifungal targets [12,13,15]. In fact, the amphotericin B-induced fungicidal effect on *C. albicans* is mediated by the Hog1 pathway [27]. In this context, enzymes involved in trehalose metabolism have been chosen as a preferential candidate to target new antifungals [14,16], since this disaccharide is absent in mammals and plays an important role as a virulence factor in prevalent pathogenic fungi, including a consistent defensive response against clinical antifungals [31]. This protective capacity of trehalose during in vivo infections has recently been extended to bacteria [37].

Although trehalose hydrolysis has received less attention with respect to the biosynthesis of disaccharide, recent evidence supports a major role of trehalases in the pathogenicity of several species of *Candida* [10,20,21,22]. In this study, we have analyzed the hypothetical toxic effect of two azoles, FLC and ITC, against a *C. parapsilosis* null mutant *atc1*Δ/*ntc1*Δ, which lacks any enzymatic detectable trehalase activity (Table 1). The correct disruption of both individual genes was also confirmed by the higher basal content of endogenous trehalose recorded in the mutant (Table 2). These azoles were chosen because the relevant *C. parapsilosis* strains display lower in vitro susceptibility to echinocandins with high MIC values [9], and azoles are recommended as preferred therapeutic antifungals [5,6,7]. Notably, although the antifungal sensitivity has not been yet determined in the single atc1Δ and ntc1Δ mutants, the double-homozygous *atc1*Δ/*ntc1*Δ null strain suppressed the resistance to oxidative and heat stress displayed by the *atc1Δ* mutant and decreased its degree of virulence [10,23].

According to our results, the involvement of trehalase enzymes in the virulence of *C. parapsilosis* should also encompass a role in resistance to antifungal treatments. Thus, the lack of a functional trehalase causes a time-course loss of viable cells induced by FLC and ITC (Figure 1), which reaches a maximum degree after 10 h of incubation, the toxic effect of ITC being higher than that of FLC (Figure 1). Further recovery (24 h) suggests that the action of both azoles is essentially fungistatic. Although our data are not entirely conclusive, they support a mechanism dependent, at least in part, on the mitochondrial respiratory pathway in the case of ITC, which is able to induce a conspicuous formation of endogenous ROS together with the increase of mitochondrial potential (Figure 2 and Figure 3). Likewise, the inability of FLC to generate a similar internal oxidative stress would explain its inability to elicit a significant degree of cell killing in *C. parapsilosis* (Figure 1, Figure 2 and Figure 3). The production of intracellular ROS triggered by Amphotericin B has been demonstrated as a universal factor of virulence in several pathogenic fungi [26], but an equivalent mechanism regarding azoles appears to be less evident, although in *Aspergillus fumigatus*, ITC is able to induce the production of mitochondrial ROS [29]. Moreover, a common pathway of oxidative-damage cellular death has been proposed to explain the fungicidal mechanism developed by distinct classes of compounds [28].

The formation of structured biofilms on indwelling devices aggravates the risk of patients from suffering clinical infections [32,33,34]. *C. parapsilosis* frequently develops persistent biofilms on intravenous catheters, which tend to withstand the exposure to AmB and azoles [38]. Remarkably, the homozygous disruption of *NTC1* and *ATC1* genes in *C. parapsilosis* itself decreases the capacity to form biofilms [23], suggesting that trehalase might be involved in the development of active biofilms. This reduction was further enhanced by the addition of the two azoles with the effectiveness of ITC being greater than that of FLC (Figure 4). The inclusion of intense oxidative exposure also induced a clear impairment in the metabolic activity of prefixed sessile cells (Figure 4). Collectively, our data support the research on central metabolic pathways as promising antifungal targets, although this proposal needs to be strengthened with evidence gathered from other compounds as well as by the screening of molecules capable of inhibiting key enzymes in fungal metabolism.

## Figures and Tables

**Table 1:** Specific activities corresponding to neutral (Ntc1p) and acid (Atc1p) trehalases measured during growth in YPD displayed by the parental strain (*Cp*) and the trehalase-deficient null mutant (*atc1*Δ/*ntc1*Δ) of *C. parapsilosis*. Identical samples were harvested and prepared at the indicated intervals, and the enzymatic activities were measured as described in Methods. Results are the mean ± SD of one representative experiment of two performed in triplicate.

Time (h)	Neutral Trehalase (Ntc1p) ^a^		Acid Trehalase (Atc1p) ^a^	
	*Cp*	*atc1*Δ/*ntc1*Δ	*Cp*	*atc1*Δ/*ntc1*Δ
1	22.5 ± 0.9	<0.3	2.7 ± 0.3	<0.3
5	18.3 ± 0.7	<0.3	3.9 ± 0.2	<0.3
10	12.7 ± 0.3	<0.3	8.1 ± 0.5	<0.3
24	6.8 ± 0.2	<0.3	9.7 ± 0.7	<0.3

^a^ Values are expressed as nmol glucose min^−1^ (mg protein)^−1^.

**Table 2:** Effect of ITC and FLC on the trehalose content on FLC- and ITC-treated cells of *C. parapsilosis*. YPD-growing exponential yeast cells (OD_600_ = −0.8 – 1.0) of the two studied strains were exposed at 37 °C for 1 h with the indicated concentrations of FLC or ITC. Trehalose content was measured as described in Methods. The data represent the mean ± SD of three independent determinations. Statistically significant differences (*** = *p* < 0.001) were recorded with respect to an untreated control according to the Mann–Whitney U test.

Treatment	Trehalose (nmol (mg wet wt)_−1_)	
	*Cp*	*atc1*Δ/*ntc1*Δ
Control	4.8 ± 0.5	3.3 ± 0.2
Fluconazole (1.0 μg/mL)	6.3 ± 0.2 ***	4.9 ± 0.2 ***
Itraconazole (0.3 μg/mL)	9.6 ± 0.3 ***	7.2 ± 0.3 ***

## Data Availability

Not applicable.

## Abbreviations

The following abbreviations are used in this manuscript:

AmB	Amphotericin B
FLC	Fluconazole
ITC	Itraconazole
PBS	Phosphate-Buffered Saline
ROS	Reactive Oxygen Species
